# Radio-resistance of hypoxic tumors: exploring the effects of oxygen and X-ray radiation on non-small lung cancer cell lines

**DOI:** 10.1186/s13014-023-02275-8

**Published:** 2023-05-12

**Authors:** Rachel Hanley, Francesca Pagliari, Daniel Garcia-Calderón, Joana Fernandes Guerreiro, Géraldine Genard, Jeannette Jansen, Clelia Nisticò, Maria Grazia Marafioti, Luca Tirinato, Joao Seco

**Affiliations:** 1 Biomedical Physics in Radiation Oncology, German Cancer Research Center (DKFZ), Im Neuenheimer Feld, Heidelberg, Germany; 2 Department of Physics and Astronomy, Heidelberg University, Im Neuenheimer Feld, Heidelberg, Germany; 3 Faculdade de Medicina Veterinária, Universidade de Lisboa, Lisbon, Portugal; 4 Experimental and Clinical Medicine Department, University Magna Graecia of Catanzaro, Catanzaro, Italy

## Abstract

**Background:**

Solid tumors are often riddled with hypoxic areas, which develops as a result of high proliferation. Cancer cells willingly adapt and thrive in hypoxia by activating complex changes which contributes to survival and enhanced resistance to treatments, such as photon radiation. Photon radiation primarily relies on oxygen for the production of reactive oxygen species to induce DNA damage. The present *in-vitro* study aimed at investigating the biochemical responses of hypoxic non-small cell lung cancer (NSCLC) cells, particularly the effects on the DNA damage repair systems contributing to more radioresistant phenotypes and their pro- and anti-oxidant potential, within the first 24 h post-IR.

**Methods:**

NSCLC cell lines (H460, A549, Calu-1) were irradiated using varying X-ray doses under normoxia (21% O_2_) and hypoxia (0.1% O_2_). The overall cell survival was assessed by clonogenic assays. The extent of irradiation (IR)-induced DNA damage was evaluated by analyzing γ-H2AX foci induction and the altered expression of repair genes involved in non-homologous end joining and homologous recombination pathways. Moreover, cell-altered responses were investigated, including the nuclear and cytosolic hydrogen peroxide (H_2_O_2_) production, as well as the associated anti-oxidant potential, in particular some components related to the glutathione system.

**Results:**

Analysis of clonogenic survival revealed an enhanced radioresistance of the hypoxic NSCLC cells associated with reduced DNA damage and a downregulation of DNA repair genes. Moreover, nuclear H_2_O_2_ levels were IR-induced in a dose-dependent manner only under normoxia, and directly correlated with the DNA double-strand breaks. However, the observed nuclear H_2_O_2_ reduction in hypoxia appeared to be unaffected by IR, thus highlighting a possible reason for the enhanced radioresistance of the hypoxic NSCLC cells. The cellular antioxidant capacity was upregulated by IR in both oxygen conditions most likely helping to counteract the radiation effect on the cytosolic H_2_O_2_.

**Conclusions:**

In conclusion, our data provide insight into the adaptive behavior of radiation-resistant hypoxic NSCLC cells, in particular their DNA repair and oxidative stress responses, which could contribute to lower DNA damage and higher cell survival rates following X-ray exposure. These findings may therefore help to identify potential targets for improving cancer treatment outcomes.

**Supplementary Information:**

The online version contains supplementary material available at 10.1186/s13014-023-02275-8.

## Introduction

Radiation therapy (RT), often combined with chemotherapy, immunotherapy and/or surgery, is a common modality used to treat malignant solid tumors. RT works by depositing energy in the cancerous tissue causing direct and indirect DNA damage. X-rays are the most widely used radiation type and are considered indirectly ionizing radiation. In fact, the secondary electrons released when photons are absorbed by atoms interact with water molecules causing radiolysis. This creates free radicals which, in the presence of oxygen (O_2_), further react with it, resulting in the production of reactive oxygen species (ROS), including H_2_O_2_. High levels of ROS in cells induce damage to cellular biomolecules and different types of DNA lesions, among which double-strand breaks (DSBs) are the most detrimental due to the complexity of efficiently repairing them. All these events contribute to generate a condition known as oxidative stress [1]. In response to this, cells can activate DNA repair mechanisms, mostly non-homologous end joining (NHEJ) and homologous recombination (HR) pathways, which induce cell cycle arrest. As a consequence, cells undergo either cell death or survival depending on the severity of the damage and the efficiency of the repair systems. Since cancer cells can have dysfunctional DNA repair and basally stronger antioxidant defense systems [2, 3], their survival to RT can be favored, which results in the development of radiotherapy resistance (RR).

The extent and the outcome of DNA damage depend on the oxygen content of the irradiated tissue. Many solid tumors are characterized by regions with different and fluctuating O_2_ levels, both in concentrations (mild *versus* severe) and in time of exposure (acute *versus* chronic). When O_2_ content decreases under the physiological levels, the resulting low O_2_ concentration is defined as hypoxia. Hypoxia is an inevitable consequence of the excessive proliferation and expansion rates of tumors, wherein the increasing distance between cells and the vasculature network does not allow to sustain the high O_2_ demand of metabolically active cancer cells with an adequate O_2_ supply. For example, in healthy lung tissue, the median oxygen percentage (~ 5.6%) is almost threefold higher than the O_2_ content found in lung cancer (~ 1.9%) [4]. Further, the disorganized and defective rearrangement of the blood vessels with the increasing tumor size can generate more hypoxic milieux. Multiple clinical studies have proven hypoxia to be associated with poor prognosis after RT in patients with different types of cancer [5–7], including NSCLC [8, 9] In particular, NSCLC, with its aggressive nature and highly metastatic ability, accounts for 85% of lung cancer patient diagnosis’ and, despite of considerable progress in the treatment options, survival remains poor [10].

Adaptive responses to hypoxia typically result in slower proliferation, changes in cell cycle distribution and in the DNA damage response (DDR) network, as well as in the antioxidant defenses, which collectively contribute to develop more aggressive cell sub-populations and, therefore, to sustain survival and resistance to cancer treatments [11–15]. These adaptations and enhanced RR highlight the potential role of O_2_ as a radiosensitizer. Hypoxic tumors, in fact, typically require a 2–threefold increase in radiation dose to induce a comparable amount of damage as oxygenated tumors. This is called the oxygen enhancement ratio (OER).

Consequently, RT needs to not only overcome the cancer cell DNA repair systems, but also the corresponding cell antioxidant defenses. However, the altered antioxidant behavior, which has a large association with the DDR [16] and can therefore play a key role in the increased radio-resistance in NSCLC, has little coverage especially in the context of hypoxic microenvironments. Understanding the behavior of adapted hypoxic cells is crucial because they represent a resistant sub-population with the potential for clonal proliferation and metastatic dissemination [17].

Therefore, the object of this study was to explore the responses of three NSCLC cell lines to different X-ray doses in severe chronic hypoxic conditions (0.1% O_2_) as compared to their counterparts cultured in standardly used atmospheric conditions (21% O_2_, hereafter referred to as normoxia) within the first 24 h post-irradiation.

This radiobiological study aimed to highlight potential common and cell-type specific features of lung tumor cells and how their altered responses in hypoxia could contribute to their ability to survive IR treatment. This is important in order to gain knowledge on the role of hypoxic microenvironments in potentially favoring tumor progression and RR.

## Materials and methods

### Cell cultures

Human NSCLC cell lines, H460, A549 and Calu-1 cells were cultured in RPMI, Ham’s F-12 K (Kaighn’s) and McCoy’s 5A media, respectively, supplemented with 10% Fetal Bovine Serum and 1% PenStrep (all from Thermo Fisher Scientific, Germany) at 37 °C with 5% CO_2_ and 21% O_2_ (normoxia) and 0.1% O_2_ (hypoxia). In hypoxia, cells were incubated in a hypoxic chamber (HC) (Sci-Tive, Baker Ruskin) at 1% O_2_ for 48 h (hrs) to adapt cells to a mild oxygen depletion. Afterwards, cells were maintained in severe hypoxia (0.1% O_2_) for a further 12 h before IR and collected at 30 min (min) (total 2.5 days in hypoxic conditions) or 24 h (total 3.5 days in hypoxic conditions) post-IR while maintaining them at 0.1% O_2_. For all the experiments in hypoxia, cells were cultured in the HC by using pre-equilibrated hypoxic media.

All cell lines were a kind gift from Dr. Ina Kurth and they were tested for mycoplasma contamination using the EZ-PCR Mycoplasma Detection Kit (Biological Industries) and authenticated by SNP-profiling [18] at DKFZ.

### Clonogenic assays

In normoxia, cells were seeded in T75 cm^2^ flasks (Greiner Bio-One, Germany) and cultured overnight. Cells were then irradiated (2, 4, 6, 8 Gy) using a MultiRad225 (Faxitron Biotics, USA) (225 kV X-rays; 0.5 mm Cu-filter) and seeded, including the control flask (0 Gy). In hypoxia, cells were seeded at 1% O_2_ and subsequently exposed to 0.1% O_2_ overnight. Cells were then irradiated with a 2-factor increase to account for the OER (4, 8, 12 and 16 Gy) and seeded at different concentrations. Colonies were fixed with 100% ethanol and stained with crystal violet. The survival fraction for each cell line was calculated using the following formula: S(D) = n(D)/N(D) × 1/PE, where N is the initial number of cells seeded, n is the number of colonies counted post-treatment and PE is the plating efficiency (Additional file 1).

### γ-H2AX foci staining

Cells were seeded on coverslips at a density of 5 × 10^4^, irradiated and exposed to 21% or 0.1% O_2_ for 30 min or 24 h post-IR. After each time-point, cells were fixed with 4% paraformaldehyde, permeabilized with 0.25% Triton-X for 5 min (all reagents from Thermo fisher Scientific). Samples were blocked using 4% Bovine Serum albumin (Sigma Aldrich), then incubated with anti-phospho-Histone H2A.X antibody (1:1000) (Sigma Aldrich; #05-636) for 1 h. After washing, samples were incubated with AlexaFluor 647 goat anti-mouse antibody (Thermo Fisher Scientific; #A-21236) for 30 min. Nuclei were counterstained with 1 μg/mL of Hoechst 33342 dye (Thermo Fisher Scientific; #62249) and the coverslips were mounted and then imaged using a confocal microscope (Zeiss LSM 710). ImageJ 1.52p software was used to quantify DSBs.

### Cell cycle

Cell cycle was analyzed on nuclei 24 h post-IR by taking advantage of the ability of Nuclear Peroxy Emerald 1 (NucPE1) to interact with DNA [19] For this type of measurements, the NucPE1 signal was acquired on the linear scale at a FACS Canto ™ II flow cytometer.

### Trypan blue exclusion assay

To assess the number of viable cells at 24 h post IR, cells were detached, washed, resuspended in PBS and 10 µL of Trypan blue solution were mixed with 10 µL of cell suspension. Then, 10 µL of this mixture was analyzed using an automated cell counter (LUNA II™, Logos Biosystems) which provides both viable (unstained) and non-viable (blue stained) cells.

### Cell death analysis

After 48 h from IR, cells and supernatants were collected, washed and re-suspended in 500 µL of cold PBS. Each sample was first acquired as unstained at FACS Canto™ II flow cytometer and immediately after, each sample was stained with 2µL/mL Propidium Iodide (PI) solution (stock 10 mg/mL) (Sigma Aldrich) and acquired. All samples were excited with a 488 nm laser and the fluorescence emission was collected at 585/42 nm. The results were analyzed by FlowJo software 8.1.

### Nuclear and cytosolic H_2_O_2_ detection

NucPE1 was used to measure the nuH_2_O_2_ levels, [19] while Peroxy Yellow 1 Methyl-Ester (PY1-ME) (ENAMINE LTD, Ukraine) was used to detect the cyH_2_O_2_. After IR, one set of samples (30 min time-point) was immediately processed, while another set (24 h time-point) was cultured in normoxia and hypoxia.

For both time-points, cells were stained with the dyes in HBSS for 20 min at 1% O_2_ for normoxia and 0.1% O_2_ in hypoxia. Subsequently, cells were washed, detached using TrypLE Express (Thermo Fisher Scientific) and nuclei were extracted using 0.1% NP-40 (Thermo Fisher Scientific) for the nuH_2_O_2_ evaluation. For the cyH_2_O_2_, cells were detached and washed. Both nuclei and cells were then acquired at a FACS Canto™ II flow cytometer (Becton Dickinson). The results were analysed by FlowJo software 8.1. More details are provided in the Additional file 1.

### GSH/GSSG assay

The ratio of reduced (GSH) and oxidized (GSSG) forms of glutathione present in samples at 30 min and 24 h post-IR was determined using the GSH/GSSG-Glo™ Assay (Promega) following the manufacturer’s instructions. The luminescence was recorded at a ClarioStar plate reader (BMG LABTECH). The GSH levels were deduced by the removal of GSSG from the total glutathione values.

### RNA isolation and quantitative real time-polymerase chain reaction (RT-qPCR)

RNA isolation and RT-qPCR were performed as described in the Additional file 1, which also includes the Primers’ list.

### Statistical analysis

Statistical analysis was carried out using Sigmaplot software (Version 14.5) and the data were graphed using the same software. All data were compared to their non-IR control for their respective oxygen conditions, unless otherwise stated. Statistical significance was assessed on the mean of three independent experiments (n = 3), unless otherwise specified, using Student’s t-tests and one-way ANOVA and only p values < 0.05 were considered statistically significant.

## Results

### Cell survival of NSCLC cell lines after X-ray treatment in normoxia and hypoxia

**Fig. 1:**
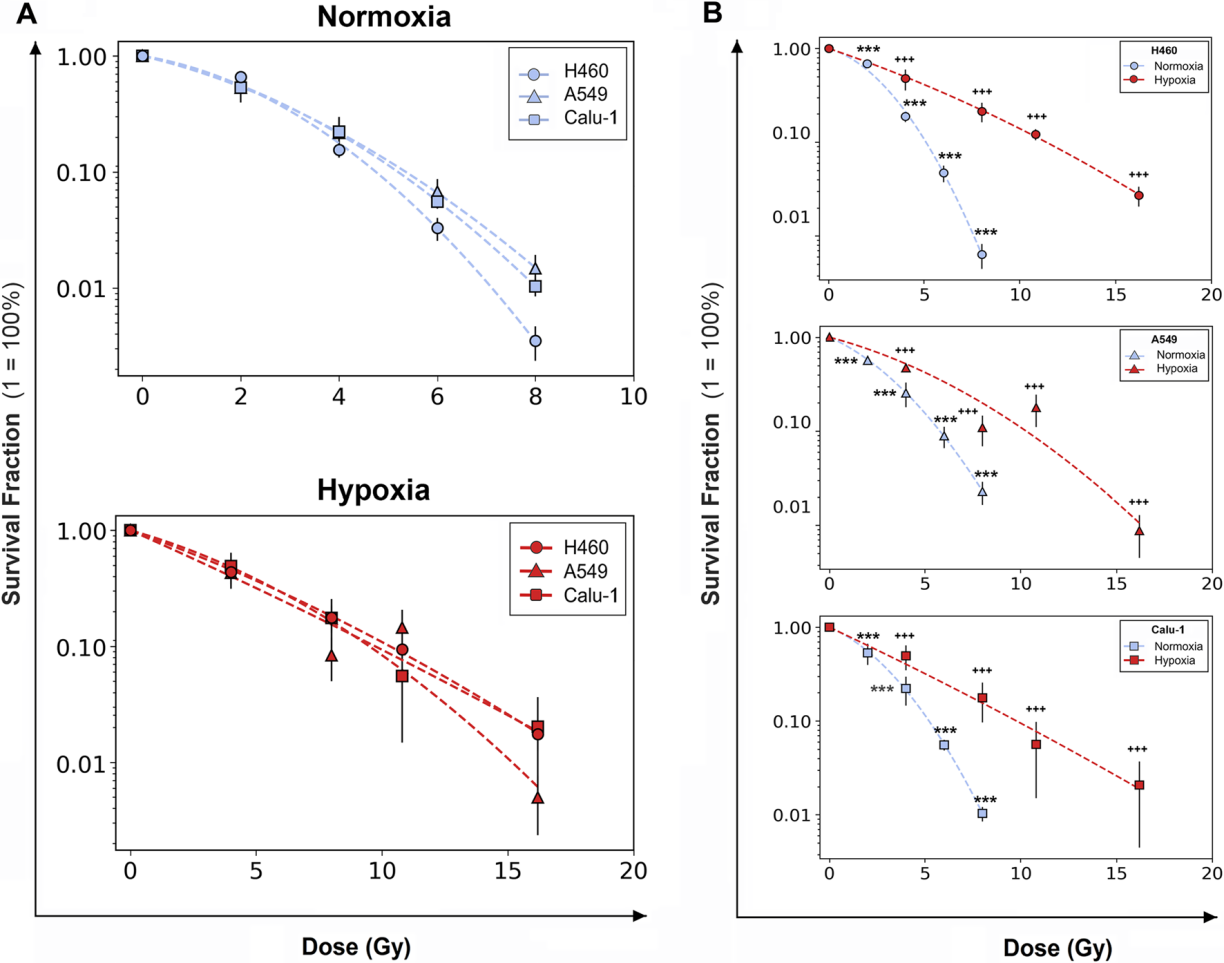
Radiosensitivity of H460, Calu-1 and A549 cells. **A** Clonogenic assays showing varying radioresistance at 21% O_2_ (upper plot) and 0.1% O_2_ (lower plot). Cell lines were irradiated with doses ranging from 2 to 8 Gy in normoxia and 4–16 Gy in hypoxia. **B** The respective survival curves separated for each cell line. Each data point is presented as mean ± SD of three separate experiments performed in quintuplicate. The survival fractions were normalized to the respective 0 Gy samples. ***p < 0.001 as compared to 0 Gy normoxia; ^+++^*p* < 0.001 as compared to 0 Gy hypoxia

The survival ability of H460, A549 and Calu-1 cells under normoxia (21% O_2_) and hypoxia (0.1% O_2_) following X-ray exposure was investigated. Cell survival fraction (SF) curves were fitted according to the linear-quadratic model (SF = exp[−(αD + βD^2^)]) [20]. In normoxia (Fig. 1A, B), the plots clearly indicated that H460 cells had the lowest cell survival ability after IR for all the doses, in comparison to the higher SF for A549 and Calu-1 cells (Additional file 2: Table 1). However, in hypoxia, the differences among the SF profiles were abolished and the cells displayed a more radio-resistant response compared to their normoxic counterparts (Fig. 1A, B and Additional file 2: Table S1). Interestingly, the hypoxic samples exhibited a general increase in the α values and a decrease in the β values (Additional file 2: Table S2), implicating a more linear curvature with loss of the quadratic component compared with the normoxic profiles. The radio-protective effects of diminished oxygen levels were further evaluated using the OER factor which was determined by calculating the ratio of doses in normoxia and hypoxia at 10% of cell survival (D10) (Additional file 2: Table S3). H460 cells had the highest OER (2.18 ± 0.179) compared to A549 (1.78 ± 0.699) and Calu-1 (1.88 ± 0.386), indicating that the removal of oxygen had a more profound effect on enhancing the RR of the more radio-sensitive oxygenated cell line. Therefore, the influence of hypoxia in cell RR and the survival of NSCLC cells was dependent on the O_2_ percentage at the time of irradiation.

### DNA DSB detection and cell repair ability after IR in normoxia and hypoxia

**Fig. 2:**
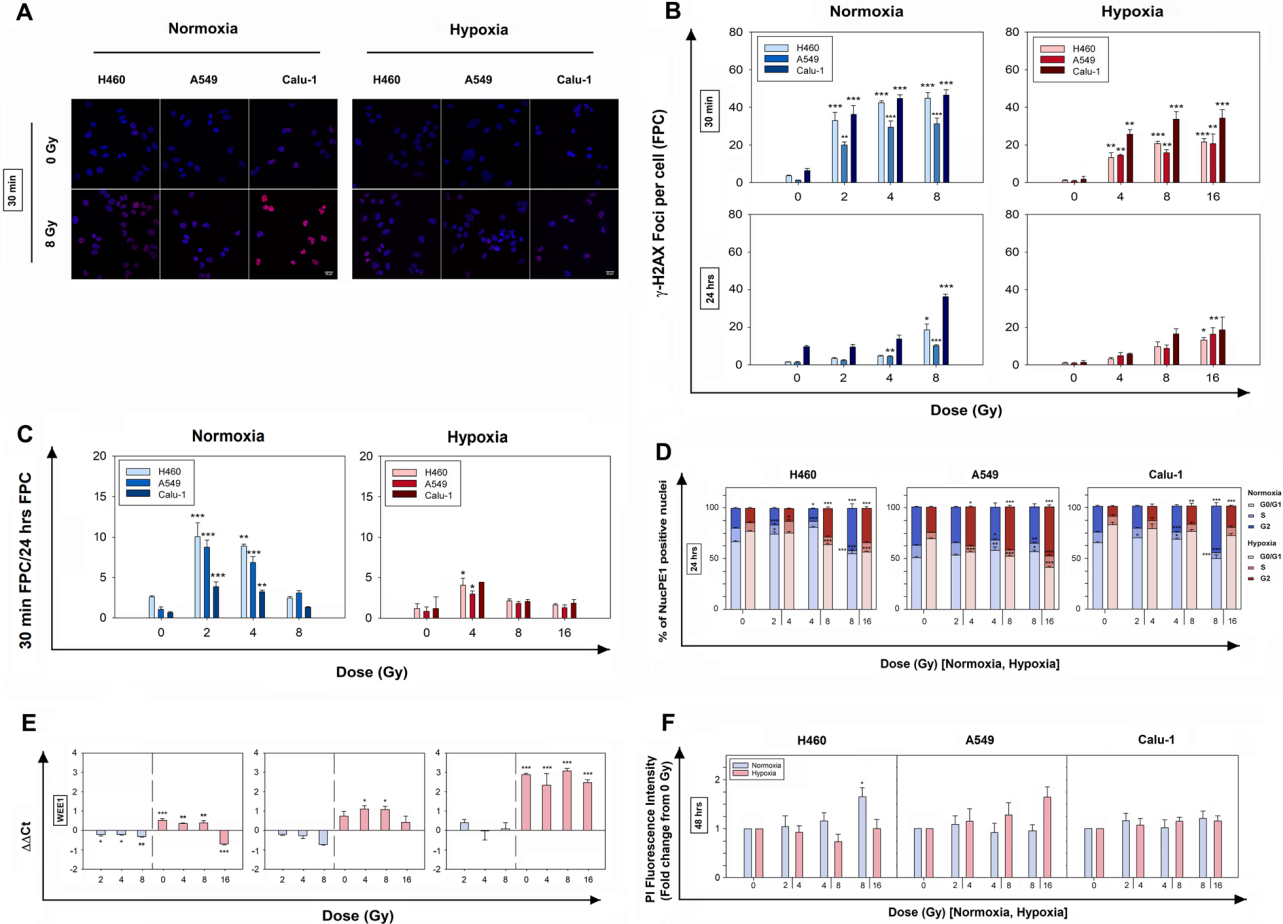
Effects of X-rays on DNA damage, gene expression, cell cycle and viability in H460, Calu-1 and A549 cells. **A** Representative confocal immunofluorescent images of γ–H2AX foci at 0 Gy and 8 Gy, 30 min after IR. **B** Detection of γ–H2AX foci in normoxia and hypoxia at 30 min and 24 h post-IR at different doses, including the respective non-irradiated controls (0 Gy). **C** Repair capacity determined by the ratio of foci count per cell (FPC) at 30 min and 24 h in normoxia and hypoxia. The data in **A** and **C** represent mean FPC ± SEM (n = 3); samples were analyzed at a confocal microscope with a sample size of > 100 cells per replicate and compared to their respective 0 Gy group (one-way ANOVA). **D** Stacked bar chart showing the cell cycle distribution (in %) of normoxic and hypoxic NucPE1-positive nuclei after 24 h from X-ray exposure. **E** Relative mRNA expression levels of WEE1 in normoxic and hypoxic samples 24 h post-IR. The data are presented as mean of the ΔΔCt values ± SEM (n = 2, each in triplicate). All samples were compared to the 0 Gy normoxic sample represented as the 0 on the x-axis (one-way ANOVA). **F** Flow cytometric quantification of dead cells 48 h post-IR by staining with propidium iodide (PI). The data in **D** and **E** represent the mean ± SEM (n = 3) and were statistically compared to their respective 0 Gy group (one-way ANOVA). *p < 0.05, **p < 0.01, ***p < 0.001

In order to understand how the oxygen level influenced cell repair capability, the initial (30 min) and residual (24 h) IR-induced γ–H2AX foci formation, which suggests accumulation of DNA damage, (Fig. 2A), was investigated. The remaining foci at 24 h can provide an indication of the repair ability of each cell line.

At 30 min post-IR in normoxia, a dose-dependent increase for all cell lines was observed in comparison with the respective non-irradiated (0 Gy) samples, with H460 and Calu-1 cells having a higher number of Foci Per Cell (FPC) (Fig. 2B). In hypoxia, the number of FPC was lower for all cell lines at 30 min post-IR, even with the OER factor applied to the doses (Fig. 2B). 24 h after X-ray exposure, the normoxic samples had repaired a considerable amount of foci in a dose-dependent manner, although for the highest doses the DNA DSBs remained significantly higher than the non-irradiated samples (Fig. 2B). This suggested that normoxic cells were able to activate the repair programs although not sufficiently for complete recovery of DNA DSBs at high doses (8 Gy). In hypoxia, the residual damage at 24 h was reduced and the dose dependency remained consistent for all cell lines (Fig. 2B). The calculated repair capacity (i.e. ratio of 30 min FPC and 24 h FPC) (Fig. 2C) reduced with increasing dose for all cell lines in both oxygen conditions, although there was a significantly lower repair ability seen in hypoxia compared with normoxia.

### Cell cycle and death post-IR in normoxia and hypoxia

Ionizing radiation induces DNA damage and consequently forces cells into cycle arrest, ultimately resulting either in cell death, if reparation is not possible, or in continued proliferation with increased genome instability. Therefore, the cell cycle profile was investigated 24 h post-IR. At 30 min, no significant changes were detectable in the cell cycle distribution most likely because of the reduced time after exposure (data not shown).

At 24 h post-IR in normoxia (Fig. 2D, and Additional file 2: Table S4), nuclei were mainly accumulated in the G0-G1 phase compared to non-irradiated samples, but at a high dose of 8 Gy a block was also visible in the G2 phase in H460 and Calu-1 cells. At 0.1% O_2_, cells resulted mainly arrested in the G2 phase at the high doses. Overall, the activation of cell cycle blocks showed intercellular variability and intra-cellular dose-dependency.

The mRNA expression of WEE1 (Fig. 2E), whose main role is preventing cells from entering mitosis too early by activating a block in the G2 or S phases [21], appeared downregulated in normoxia for H460 and A549 cells post-IR, while no significant changes were detectable in Calu-1 cells. WEE1 expression in hypoxia was instead predominantly increased (except in some cases at high doses) across cell lines which was in accordance with the hypoxic cell cycle profiles. This indicated a potential WEE1 involvement in favoring the G2 block, although the O_2_ reduction appeared to be the main factor influencing WEE1 expression compared to IR.

Moreover, the possibility that cells could undergo cell death after cycle arrests was tested 24 and 48 h after X-ray exposure by measuring the amount of dead cells and counting cells. These viability results (Fig. 2F and Additional file 3: Fig. S1 and Additional file 4: Fig. S2) showed no statistically significant changes in the dead or dying cell pool up to 48 h in both oxygen conditions (except for the H460 cells at the highest dose). This suggested that either the cells were arrested, yet viable at these time-points, or recovered from blocking, but in both cases radiation treatment did not result in cell death up to 48 h from IR.

### DNA damage repair gene expression in normoxia versus hypoxia 24 h post-IR

**Fig. 3:**
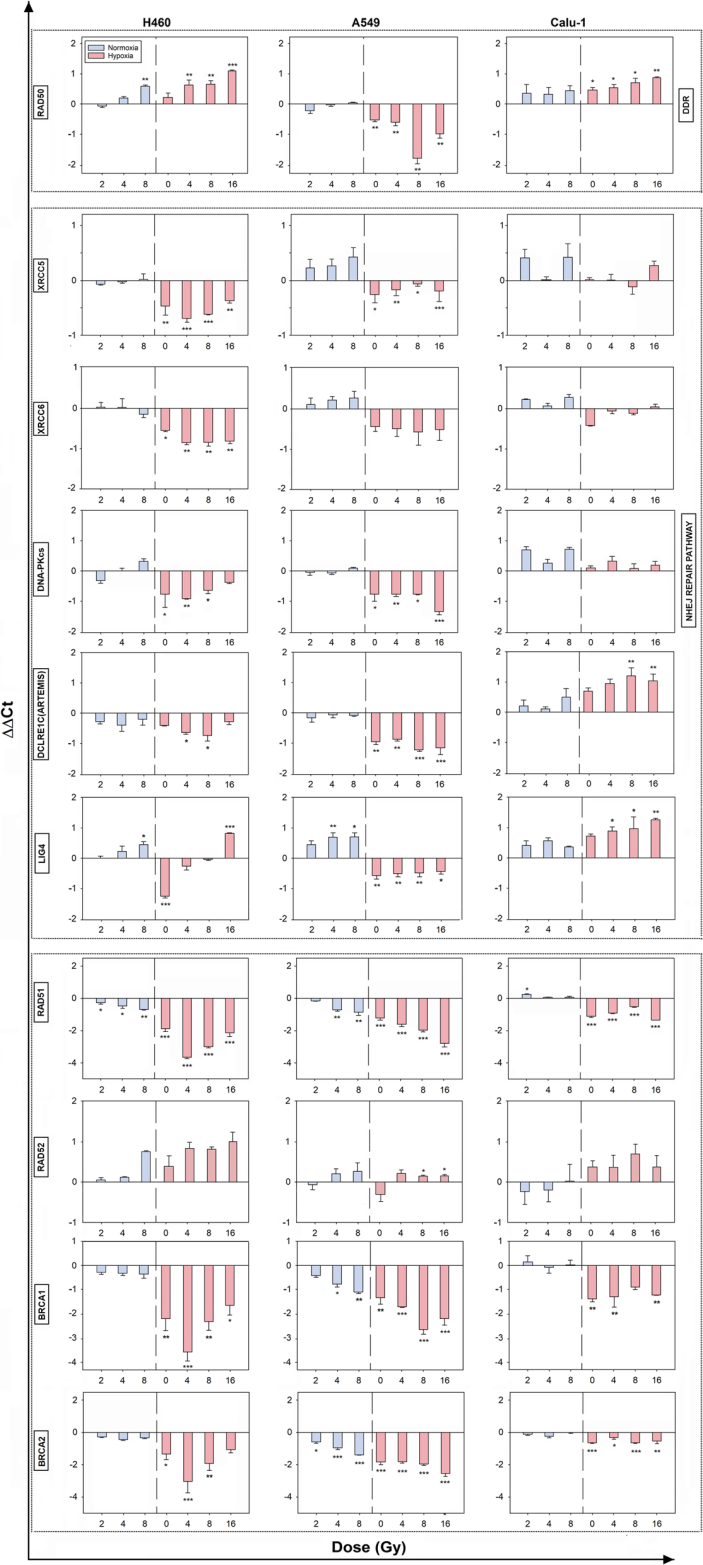
Effects of X-rays and O_2_ levels on the expression of DNA-damage response genes. Relative mRNA expression levels of RAD50, XRCC5, XRCC6, DNA-PKcs, RAD51, RAD52, BRCA1 and BRCA2 in normoxic and hypoxic samples 24 h post-IR. The data are presented as mean of the ΔΔCt values ± SEM (n = 2, each in triplicate). All samples were compared to the 0 Gy normoxic sample represented as the 0 on the x-axis (one-way ANOVA)

Based on these previous observations, the mRNA expression of some genes involved in the DDR sensory machinery (RAD50), NHEJ (XRCC5, XRCC6, DNA-PKcs, DCLRE1C and LIGASE4) and HR (RAD51, RAD52, BRCA1 and BRCA2) repair pathways was analyzed 24 h post-IR (Fig. 3). Data revealed that in all cell lines, the fluctuations of XRCC5 and XRCC6 (also known as KU80 and KU70, respectively), DNA-PKcs, DCLRE1C (also known as ARTEMIS) and LIGASE4 (LIG4) expressions in normoxia were not statistically significant, except LIG4 at the highest dose in H460 and A549 cells. At 0.1% O_2_, the NHEJ-related genes were downregulated for H460 and A549 cells with the exception of LIG4 that showed a trend to increase in H460 cells. In Calu-1 cells instead, DCLRE1C and LIG4 were both up-regulated. On the contrary, RAD51 and RAD52 showed similar trends among the cell lines in both O_2_ conditions in, albeit not always significant, with a more pronounced downregulation in hypoxia for RAD51, which was evident also for hypoxic Calu-1. BRCA1 and BRCA2 resulted both downregulated in all cell lines. Therefore, in severe hypoxic conditions, the IR doses played different roles depending on the cell lines and the two repair pathways were in general less expressed, with the exception of Calu-1 cells in which some upregulation was still detectable at 24 h from IR.

### Nuclear and cytosolic H_2_O_2_ levels post-IR in normoxia and hypoxia

**Fig. 4:**
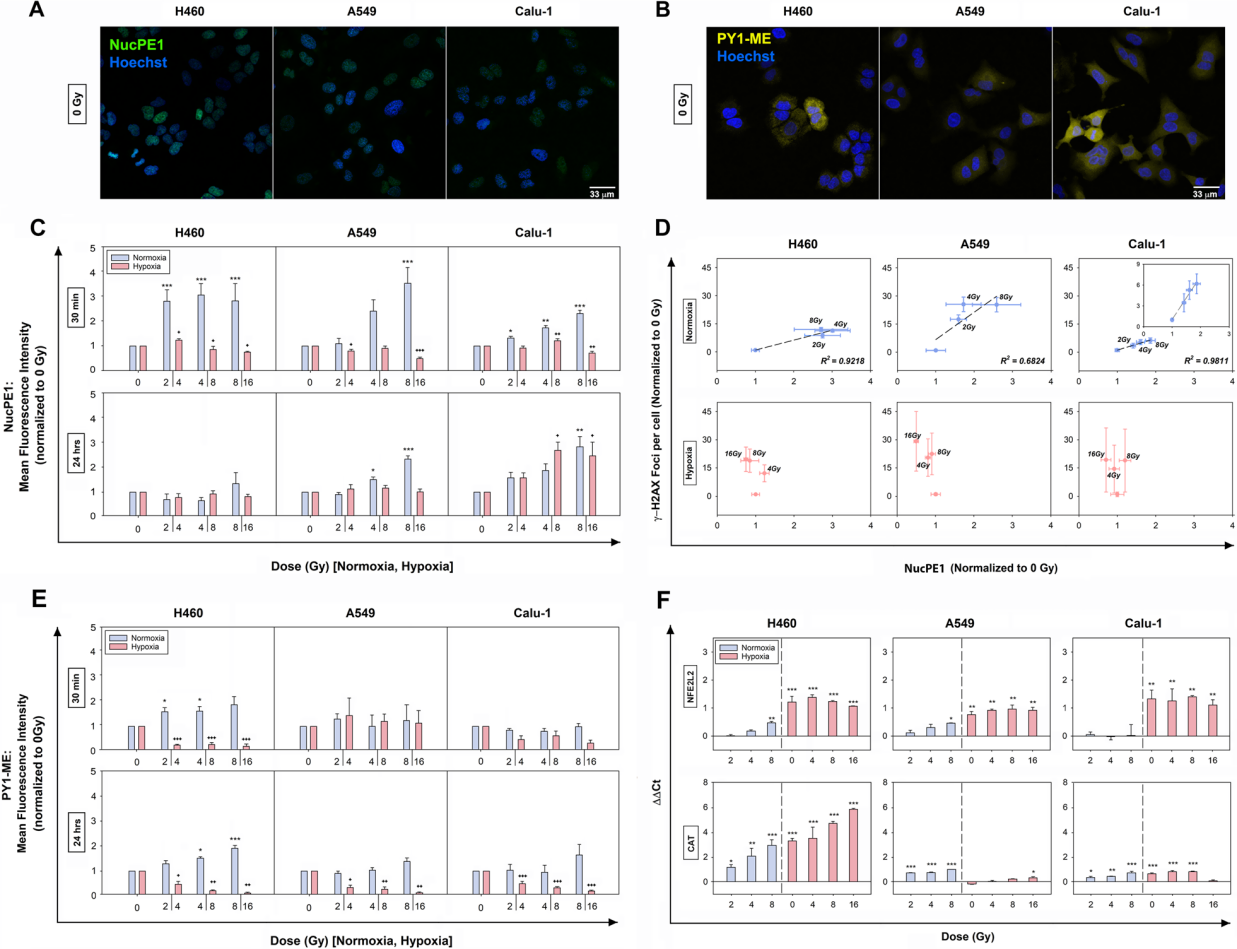
Effects of the combined hypoxia and X-ray treatments on H_2_O_2_ production and the antioxidant responses. **A** and **B** Representative confocal images of nuH_2_O_2_ and cyH_2_O_2_ detection in normoxic non-irradiated NSCLC cells by staining with NucPE1 and PY1-ME, respectively. Nuclei (blue) were counterstained with Hoechst 33,342. **C** and **D** Relative estimation of nuH_2_O_2_ and cyH_2_O_2_ content at 30 min and 24 h post-IR in normoxia and hypoxia. All samples were normalized and statistically compared to their respective normoxic or hypoxic control (0 Gy). Error bars are represented as SEM (n = 3) (one-way ANOVA). **E** Correlation between normalized (to 0 Gy) DSBs per cell and nuH_2_O_2_ levels for each dose in normoxia and hypoxia measured at 30 min post-IR. **F** Relative mRNA expression levels of intracellular NFE2L2 and CAT genes evaluated using RT-qPCR at 24 h following IR treatment in normoxia and hypoxia. The data are presented as mean of the ΔΔCt values ± SEM (n = 2, each in triplicate) (one-way ANOVA). *p < 0.05, **p < 0.01, ***p < 0.001 as compared to 0 Gy normoxia; ^+^*p* < 0.05, ^++^*p* < 0.01, ^+++^*p* < 0.001 as compared to 0 Gy hypoxia

To qualitatively estimate the nuH_2_O_2_ and cyH_2_O_2_ production after IR and its modulation in normoxia and hypoxia, NucPE1 and PY1-ME were used. Confocal images (Fig. 4A, B) confirmed the preferential localization of NucPE1 and PY1-ME in the nuclei and cytosol, respectively, of all cell lines. Moreover, to reduce the possibility of cytosolic background fluorescence or artifacts, the NucPE1 signal was measured only on stained nuclei extracted after IR.

The H_2_O_2_ measurements at 30 min post-IR aimed at investigating the initial IR-induced H_2_O_2_ levels and potentially correlating nuH_2_O_2_ with the DSB damage. Further, the H_2_O_2_ content analyzed 24 h post-IR aimed at evaluating cell ability to counteract the oxidative stress induced by IR treatments.

Results obtained from the normoxic samples at 30 min indicated a fast rise in nuH_2_O_2_ production with increasing doses for A549 and Calu-1, whereas for H460 cells similar increases were produced for all doses with a clear plateau of the profile (Fig. 4C and Additional file 5: Fig. 3A). At 24 h post-IR, nuH_2_O_2_ levels returned to basal values in H460 cells, whereas they remained higher than the controls in A549 and Calu-1 cells for 4 and 8 Gy. By reducing oxygen availability, the effects of radiation after 30 min on nuH_2_O_2_ were less pronounced (Fig. 4C and Additional file 5: Fig. 3A) and no dose dependency was observed for all cell lines. At 24 h, changes in the nuH_2_O_2_ production were no longer detectable, except for the highest doses in Calu-1 cells (Additional file 2: Table 5). With the exception of Calu-1 cells at 30 min, all the hypoxic cells showed a minor content of nuH_2_O_2_ compared to the normoxic samples, which was more evident at 24 h post-IR (Additional file 5: Fig. 3A).

Interestingly, by plotting the DSB data against the nuH_2_O_2_ data obtained 30 min post-IR (Fig. 4D), a linear dependence of the DSBs produced with nuH_2_O_2_ was present in normoxia. For the hypoxic cells instead, no correlation was observed (Fig. 4D).

The cyH_2_O_2_ levels of normoxic cells were seemingly less responsive to IR compared to the nuH_2_O_2_ levels (Fig. 4E and Supplementary Fig. 3B). In fact, at both time-points, only normoxic H460 cells appeared to increase their cyH_2_O_2_ content. On the contrary, in hypoxic cells, the cyH_2_O_2_ levels were subjected to a decrease after IR at both time-points, except for A549 cells at 30 min (Additional file 2: Table 6). Moreover, also the cyH_2_O_2_ levels were reduced compared to the normoxic cells, albeit H460 cells at 0 Gy showed a higher content (Additional file 5: Fig. 3B). This apparent discrepancy at the moment remains to be better investigated.

In general, results potentially suggested a lower oxidative stress in hypoxia and a more readily active and sustained antioxidant capacity in the cytoplasm compared to the nucleus.

Based on these findings, the relative mRNA expression levels of the antioxidant response master regulator, NFE2L2 (also known as NRF2), were analyzed 24 h post-IR (Fig. 4F). At 21% O_2_, in H460 and A549 cells a dose-increase was mainly observed which resulted significant only for the highest dose. In normoxic Calu-1 cells, no modulation of NFE2L2 gene expression was detectable at any dose. However, the reduction of O_2_ markedly induced the overexpression of NFE2L2 in all cell lines independently on the IR doses. The analysis of mRNA levels of CAT, which is induced by NFE2L2 and is directly involved in H_2_O_2_ removal, showed IR-induced stimulation in normoxia for all cell lines (Fig. 4F). In hypoxia, CAT mRNA was upregulated in H460 and Calu-1 cells, with a drop at 16 Gy for the latter cell line. In A549 cells, a significant change in CAT mRNA expression was observed only at 16 Gy, even though NFE2L2 was upregulated, suggesting that NFE2L2 was not directly influencing CAT gene expression in this cell line. However, a mild tendency towards an increased expression was observed following irradiation in hypoxia.

These findings suggested that IR triggered some antioxidant responses which were noticeably influenced by the O_2_ level.

### Measurements of glutathione levels after X-rays in normoxia and hypoxia

**Fig. 5:**
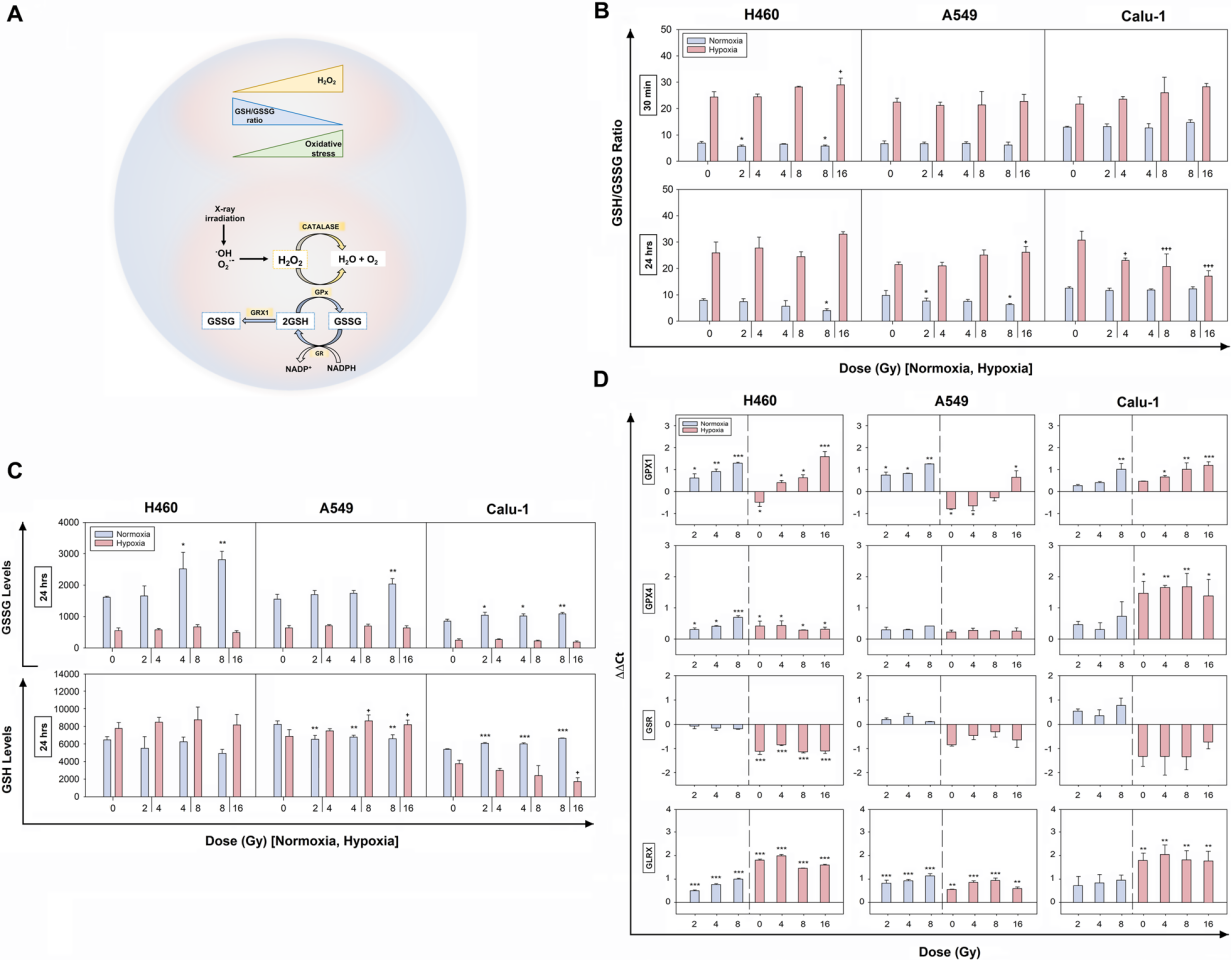
Effects of the IR-induced oxidative stress on the glutathione system in normoxia and hypoxia**.** **A** Schematic representation of some key components of the glutathione system involved in H_2_O_2_ detoxification. **B** Ratio of GSH/GSSG levels in normoxic and hypoxic samples at 30 min and 24 h post-IR in H460, A549 and Calu-1 cells. **C** Oxidized (GSSG) and reduced (GSH = total glutathione—GSSG) glutathione levels at 24 h post-IR following incubation in normoxia and hypoxia. In **B** and **C** the data are showed as mean ± SD (n = 3, in duplicate) and were statistically compared to their respective 0 Gy (one-way ANOVA). **D** Relative mRNA expression levels of GPX1, GPX4, GSR, GLRX genes at 24 h post-IR. Data are presented as the ΔΔCt mean ± SEM (n = 2, each in triplicate) (one-way ANOVA). *p < 0.05, **p < 0.01, ***p < 0.001 as compared to 0 Gy normoxia; ^+^*p* < 0.05, ^++^*p* < 0.01, ^+++^*p* < 0.001 as compared to 0 Gy hypoxia

In order to investigate cellular ability to scavenge H_2_O_2_, measurements of intracellular reduced and oxidized glutathione levels were also performed. Glutathione, in fact, is the most abundant low-molecular-mass antioxidant serving as an essential cofactor for the reduction of H_2_O_2_ to H_2_O catalyzed by glutathione peroxidases (GPX). The ratio of GSH/GSSG is high under normal conditions and decreases upon pro-oxidant stresses providing an indication of the cellular redox environment (Fig. 5A).

In normoxia, changes in the GSH/GSSG ratio at 30 min were observed only in H460 cells with a slight reduction at 2 and 8 Gy and such a profile was maintained, although slightly decreased, up to 24 h (Fig. 5B). A similar trend at the later time-point was observed for normoxic A549, suggesting a condition of mild oxidative stress in both these cell lines. However, the strongest difference in the GSH/GSSG ratios was observed in hypoxic samples compared to their normoxic counterparts (Fig. 5B), which were maintained higher also after the X-ray exposure at both time-points, with only a decreased trend in Calu-1 cells. In this latter cell line, at 24 h, the GSH/GSSG reduction was dose-dependent and associated with a GSH depletion (Fig. 5C). Instead, in the other two cell lines, the GSSG hypoxic levels were maintained at a lower level than in normoxia (Fig. 5C). This suggested a stronger ability to cope with the IR-induced intracellular oxidative stress in comparison to Calu-1 cells.

Altogether, this evidence indicated that the main role in modulating the glutathione ratio was played by the oxygen levels and the radiation treatment exhibited only cell specific effects.

In order to assess whether the observed effects were correlated with a modulation of the glutathione system genes, the mRNA expression of GPX1 and 4, Glutathione-disulfide Reductase (GSR), and Glutaredoxin-1 (GLRX) was evaluated (Fig. 5D). The results showed an upregulation of GPX1 mRNA post-IR in normoxic samples to a different extent in all cell lines. Under hypoxia, cell-type specific behaviors were observed with downregulations in H460 and A549 cells, and upregulations in Calu-1 cells. When irradiated in hypoxia, all cell lines responded with an increased gene expression compared to their respective untreated hypoxic controls. However, in comparison with the 0 Gy normoxic samples, in H460 cells GPX1 expression was upregulated already at 4 Gy, in A549 cells only the highest dose led to an upregulation, and for Calu-1 cells GPX1 was always overexpressed. Instead, GPX4 mRNA levels were upregulated in all cell lines, although not always significantly. Also, GSR mRNA levels were not statistically modified in normoxia and were observed downregulated in hypoxia. In contrast, GLRX mRNA levels were always upregulated in all cell lines, with a slight dose-dependency in normoxia and a larger effect following O_2_ reduction for H460 and Calu-1 cells compared to A549 cells. Therefore, in hypoxia, GLRX rather than GSR could mostly participate in maintaining the low levels of GSSG observed in the present study.

## Discussion

The high NSCLC RR results in reduced treatment success rates [22] which is especially problematic when hypoxic regions develop. In these areas, cells activate adaptive responses characterized by altered redox and energy metabolisms, as well as repair mechanisms, all contributing to RR and intratumor response heterogeneity [22]. In this study, we show that three NSCLC lines displayed different radiosensitivity in normoxia, with H460 being the most radiosensitive. Conversely, the reduced availability of O_2_ abolished such differences and made the survival profiles more similar to each other. Moreover, all three cell lines showed higher RR in hypoxia, which is in agreement with the data reported in literature [23, 24].

Hypoxia induced varying “protectiveness” among the investigated NSCLC lines, as indicated by their OER factors, suggesting differing molecular mechanisms which aid in their survival following IR and exposure to low oxygen conditions. Survival of irradiated cells is linked to the amount of radiation-induced DNA DSBs produced and the ability of cells to properly repair them. We observed that a DNA damage-dose response was obtained both in normoxia and hypoxia, albeit with much lower OER-corrected FPC in hypoxia. This strengthens the concept that the reduced levels of O_2_ confer protection against radiation-induced DNA damage soon after IR [25]. Moreover, residual DNA damage was considerably reduced after 24 h, indicating sufficient repair ability, which was higher in normoxia than in hypoxia. This could be related either to the lower initial DSBs induced in hypoxic conditions and thereby only minimally stimulating the repair systems, or to a more functional repair signaling in normoxia within the first hours after IR. In this regard, such repair ability did not correlate with a higher repair gene expression at 24 h post-IR. This suggested that in normoxia, at a gene level, the main players of the NHEJ (XRCC5 and XRCC6 involved at the beginning, followed by DNA-PKcs and later by DCLRE1C and finally by LIG4) and HR already returned to basal gene level expression or, in some cases, were even down-regulated.

On the contrary, a cell-type specificity, as opposed to a clear dose-dependency, was observed in hypoxia. In fact, in hypoxia the O_2_ reduction was already able to reduce the expression of the majority of the investigated genes. Interestingly, in spite of the lower DSB induction and a general repair gene down-regulation in hypoxia, H460 and Calu-1 cells showed a higher expression of RAD50 with a slight dose-dependency. RAD50 is part of the MRN complex, which is crucial at the beginning of DNA damage induction being an early sensor of DSBs and probably directing the repair choice towards the NHEJ or the HR routes. Moreover, in Calu-1 cells DCLRE1C and LIG4 were also upregulated at 24 h post-IR. DCLRE1C and LIG4 participate in the last steps of the DNA repair. All these upregulations in Calu-1 cells might be correlated with the higher levels of basal and especially residual DSBs.

The evidence that hypoxic cells mainly downregulate NHEJ and HR pathways was also described by other groups, although some controversies still remain [26–32]. However, these data clearly showed a strong differential behavior between normoxic and hypoxic cells, indicating that the O_2_ levels mostly influenced gene expression.

It is believed that hypoxia-mediated downregulation of the DNA repair mechanisms can induce accumulation of unrepaired lesions and further lead to genomic instability associated with more resistant phenotypes [17, 27]. In this study, however, although the repair genes were under-expressed, most IR-induced DNA damage were repaired after 24 h in both oxygen conditions. This raised the open question of how a reduced DNA repair ability could lead to decreased levels of DNA damage and eventually increased RR, as shown in the clonogenic assays.

Additionally, the IR-triggered repair mechanisms work in a cell cycle-dependent manner by blocking cells at specific checkpoints [33–35]. After 24 h, in normoxia most cells accumulated in the G0/G1 phase, while in hypoxia in the G2 phase, suggesting the capability of delaying the cell cycle thereby giving cells more time to repair the DNA lesions or to activate cell death programs. In this respect, cell viability was unaffected until 48 h post-IR. Nevertheless, this cannot exclude the possibility that cells entered a senescence state or activated cell death programs later than the time-points here considered. This, however, was not analyzed in this study as our main interest was to investigate cell responses within 24 h. Therefore, it might be possible that the activation of alternative repair pathways and cellular survival strategies in our hypoxic conditions might be responsible for the repair ability observed in the present study.

It should be noted that different genetic backgrounds most likely are also playing a big role in influencing cell responses to IR and cellular long-term survival ability. In particular, we suspect that the high basal level of γ–H2AX foci seen in Calu-1 cells could be partly attributed to the lack of the TP53 protein. Therefore, Calu-1 cells might bypass G0/G1 checkpoint and accumulating damage and mutations. This might also explain some up-regulations of the repair genes, such as RAD50 in hypoxia, in this cell line. Several studies highlight the importance of TP53 gene in instigating a balance between the genes relevant for the two repair pathways and can further elucidate a possible reason for the radioresistance of Calu-1 cells, albeit the high basal γ–H2AX foci. Moreover, Calu-1 cells are mutant KRAS (G12C). KRAS controls cell cycle progression after irradiation and KRAS is maintained in a continuous active state in mutated cells. Preclinical studies showed that DNA repair systems are affected in mutant KRAS [36] and cell survival is increased after irradiation in NSCLC [37–39]. KRAS mutation increases the expression of WEE1, which in our study was particularly pushed in hypoxic Calu-1 cells [36]. However, H460 and A549 cells also harbor KRAS mutations (Q61H and G12S, respectively) but they are TP53-wt [41, 42]. In particular, KRAS mutation in A549 has been shown to induce less DSBs post-IR and favour their radioresistance [37]. KRAS is involved in various signaling pathways, including AKT/mTOR, MAP-kinase and Ral pathways, and therefore the downstream effectors of KRAS are multiple and might be differently activated in these three cell lines.

Moreover, in A549 cells, inactivation of the tumor suppressor CDKN2A gene, which encodes for p16INK4α, p14ARF and p12 proteins, is present. These proteins induce cell cycle arrest in G1 and G2 by preventing TP53 degradation. Therefore, in A549 cells, albeit TP53 is not mutated as in Calu-1 cells, its functions might be partially compromised, thus reducing cell radiosensitivity.

Altogether, these genetic fingerprints most likely participate in influencing the different long-term survival of these cell lines observed in normoxia and underline the importance of intrinsic factors in each cell line which exert different effects on the pathogenesis, progression and prognosis of NSCLC. However, it appears evident that the mechanisms behind hypoxia-induced RR, where cells showed similar survival abilities, are unique, yet complex and multifactorial, and therefore require further research.

One of the main causes of IR-induced DNA damage is attributed to H_2_O_2_ production, which we showed to be cell-type and subcellular compartment specific, as well as oxygen-dependent. A dose dependency of the IR-induced nuH_2_O_2_ could only be observed soon after IR and when oxygen, necessary for the chemical reactions to occur, was present. Interestingly, the linear dependency between nuH_2_O_2_ and DNA DSBs following increasing X-ray doses in normoxia provides unique evidence of a potential causal relation between nuH_2_O_2_ production and DNA damage induction soon after IR in oxygenated cells. Contrarily, the absence of a trend in hypoxia indicated that causality cannot be assumed, reiterating the important role played by the O_2_ in favoring IR-induced DNA damage.

In general, the mechanisms behind the antioxidant responses appeared to be triggered differently in a cell-specific manner by the combined O_2_- and IR-treatments. The low cyH_2_O_2_ in all hypoxic irradiated cell lines at 24 h post-IR indicated a low level of cytoplasmic oxidative stress which was in accordance with the high GSH/GSSG ratios in irradiated H460 and A549 cells, and with the upregulation of GPX4 mRNA and, for some samples, CAT mRNA. Hypoxic Calu-1 cells were instead the only cells showing a dose-dependent reduction of the GSH/GSSG ratios 24 h post-IR. In this cell line, we also observed that nuH_2_O_2_ tended to increase after 24 h, which might at least partially explain the increased oxidative stress compared to the other cell lines. In general, glutathione is crucial for cell survival and, although mainly localized in the cytosol, it is also present in the nucleus [42–44]. Therefore, the high levels observed in chronically exposed hypoxic cancer cells, and the common associated upregulation of NFE2L2 (i.e. master gene driving antioxidant responses), might both contribute to the low oxidative stress observed in the cytosol of irradiated cells and to some extent control the nuH_2_O_2_. Further, the down-regulation of the repair genes in hypoxia could be linked to the low level of oxidative stress, since several studies have shown that oxidant injury, including H_2_O_2_, was able to influence DNA repair responses [16].

It is worth highlighting that NFE2L2 is often found upregulated and associated with poor prognosis in NSCLC patients [45]. In particular, data have shown that NFE2L2 activation prevents oxidative stress-induced cell death by increasing GSH metabolism, as well as promoting tumor aggressiveness and RR [46, 47]. Therefore, the strong upregulation of NFE2L2 observed in our study, which was unaffected by IR, might be central to the RR of hypoxic cells, by driving a set of responses which permits them to adapt to hypoxic environments as well as to counteract the effects of the treatments. Instead, GPX1 gene expression appeared to be IR-sensitive and cell-type specific, suggesting that IR-treatments did in fact further stimulate the antioxidant defenses in hypoxic cells. Of note, our results also show that non-irradiated hypoxic cells displayed lower levels of oxidative stress compared to normoxic samples, as assessed by reduced/oxidized GSH assay. However, several published data have reported an increase of ROS production in hypoxic conditions [48, 49], albeit the well-accepted argument that hypoxic cells have lower metabolic rates which should therefore result in lower oxidative stress, as also reported by other studies [49]. The reasons of this apparent discrepancy are still unclear, but they may reflect the involvement of other radical and non-radical species, which were not considered in the present study. Moreover, the O_2_ levels used in different studies, the acute versus chronic exposure and the handling of hypoxic samples during the analyses can affect cellular responses and might contribute to the diverse data reported.

Additionally, since the expression of GSR, requested to regenerate GSH (Fig. 4A), is inducible upon oxidative stress, its reduction in hypoxia, with no predominant effects exerted by X-ray doses, could be due to the high GSH/GSSG ratios as a possible inhibitory loop, which remains to be proven. Instead, the levels of GLRX, which is part of the GSH-related enzymes (Fig. 4A) and has been linked to a decrease in ROS and improved survival [50–52] were generally upregulated in hypoxia and after IR. However, while GLRX function in cancer under normoxic conditions has been well investigated, its role in severe hypoxic microenvironments and upon IR-treatments remains to be fully explored, and here we provide the first evidence for its possible involvement in cellular responses under hypoxia and following photon radiation.

## Conclusions

The findings here reported indicate that hypoxia greatly, contributed to radioresistance of cancer cells derived from the same organ as compared to their normoxic counterparts, although a cell-type heterogeneity was observed at some extent. Lung cancer cells which have adapted to severe hypoxia showed consistent intracellular antioxidant abilities, which could efficiently and promptly control the IR-induced H_2_O_2_, thus contributing to the hypoxically induced RR. Of note, this study shows that low O_2_ levels over the radiation doses seemed to play the main role in influencing cell responses and in causing the strongest differences in cell behavior.

Although further and detailed studies are requested to unveil the molecular mechanisms underlying the evidence shown here, the present work offers insights into the biochemical responses and the changes in the gene expression patterns of NSCLC cells exposed to severe and prolonged hypoxia after radiation therapy. Such observations might help to better elucidate the behavior of resistant cancer cells and in the future provide potential targets for improving cancer treatment outcomes.

## Supplementary Information

**Additional file 1**. Supplementary Materials and Methods.

**Additional file 2**. Additional Tables 1-6 reporting values of cell survival fractions, alpha and beta, doses at 10% of cell survival, Cell cycle data, NucPE1 and PY1-ME fluorescence signals.

**Additional file 3**. Fig. 1: Representative histograms of cell death after IR in normoxia and hypoxia as assessed by Propidium Iodide staining and subsequent FACS analysis

**Additional file 4**. Fig. 2: Cell growth at 24 hrs after IR in normoxia and hypoxia as assessed by Trypan blue assay. Data are shown as mean±SD.

**Additional file 5**. Fig. 3: Relative estimation of nuH_2_O_2_and cyH_2_O_2_content by staining cells with NucPE1 and PY-1ME probes at 30 min and 24 hrs post-IR in normoxia and hypoxia. All samples were normalized and statistically compared to the normoxic control. Error bars are represented as SEM. *p<0.05, **p<0.01, ***p<0.001

## Data Availability

All data generated not included in this study and in its Additional Information f Files are available and will be shared upon request to the corresponding authors.

## Abbreviations

BRCA1: Breast cancer gene 1
BRCA2: Breast cancer gene 2
CAT: Catalase
DDR: DNA damage response
DNA-PKcs: DNA-activated, protein kinase, catalytic subunit
DCLRE1C (alias ARTEMIS): DNA cross-link repair 1C
FPC: Foci per cell
IR: Irradiation
GPX: Glutathione peroxidase
GSR: Glutathione reductase
GLRX: Glutaredoxin-1
GSH: Reduced glutathione
GSSG: Oxidized glutathione
HR: Homologous recombination
NFE2L2 (alias NRF2): Nuclear factor erythroid 2-related factor 2
NSCLC: Non-small cell lung cancer
NHEJ: Non-homologous end joining
NucPE1: Nuclear peroxy emerald 1
OER: Oxygen enhancement ratio
PY1-ME: Peroxy yellow 1-methyl-ester
ROS: Reactive oxygen species
RR: Radiotherapy resistance
XRCC5 (alias KU80): X-ray repair cross complementing 5
XRCC6 (alias KU70): X-ray repair cross complementing 6

## References

[1] Perillo B, Di Donato M, Pezone A (2020). ROS in cancer therapy: the bright side of the moon. Exp Mol Med.

[2] Hayes JD, Dinkova-Kostova AT, Tew KD (2020). Oxidative stress in cancer. Cancer Cell.

[3] da Motta LL, De Bastiani MA, Stapenhorst F, Klamt F (2015). Oxidative stress associates with aggressiveness in lung large-cell carcinoma. Tumor Biol.

[4] McKeown SR (2014). Defining normoxia, physoxia and hypoxia in tumours - implications for treatment response. Br J Radiol.

[5] Horsman MR, Overgaard J (2016). The impact of hypoxia and its modification of the outcome of radiotherapy. J Radiat Res.

[6] Richards CH, Mohammed Z, Qayyum T (2011). The prognostic value of histological tumor necrosis in solid organ malignant disease: a systematic review. Future Oncol.

[7] Nordsmark M, Bentzen SM, Rudat V (2005). Prognostic value of tumor oxygenation in 397 head and neck tumors after primary radiation therapy. An international multi-center study. Radiother Oncol.

[8] Brustugun OT (2015). Hypoxia as a cause of treatment failure in non-small cell carcinoma of the lung. Semin Radiat Oncol.

[9] Salem A, Asselin MC, Reymen B (2018). Targeting hypoxia to improve non-small cell lung cancer outcome. J Natl Cancer Inst.

[10] Knight SB, Crosbie PA, Balata H (2017). Progress and prospects of early detection in lung cancer. Open Biol.

[11] Muz B, de la Puente P, Azab F, Azab AK (2015). The role of hypoxia in cancer progression, angiogenesis, metastasis, and resistance to therapy. Hypoxia.

[12] Thomlinson RH, Gray LH (1955). The histological structure of some human lung cancers and the possible implications for radiotherapy. Br J Cancer.

[13] Zhang L, Hill RP (2004). Hypoxia enhances metastatic efficiency by up-regulating Mdm2 in KHT cells and increasing resistance to apoptosis. Cancer Res.

[14] Vaupel P (2008). Hypoxia and aggressive tumor phenotype: implications for therapy and prognosis. Oncologist.

[15] Harada H (2016). Hypoxia-inducible factor 1–mediated characteristic features of cancer cells for tumor radioresistance. J Radiat Res.

[16] Srinivas US, Tan BWQ, Vellayappan BA, Jeyasekharan AD (2019). ROS and the DNA damage response in cancer. Redox Biol.

[17] Bristow RG, Hill RP (2008). Hypoxia, DNA repair and genetic instability. Nat Rev Cancer.

[18] Castro F, Dirks WG, Fähnrich S (2013). High-throughput SNP-based authentication of human cell lines. Int J Cancer.

[19] Dickinson BC, Tang Y, Chang Z, Chang CJ (2011). A nuclear-localized fluorescent hydrogen peroxide probe for monitoring Sirtuin-mediated oxidative stress responses in vivo. Chem Biol.

[20] Kellerer AM, Rossi HH (1978). A generalized formulation of dual radiation action. Radiat Res.

[21] Ghelli Luserna Di Rorà A, Cerchione C, Martinelli G, Simonetti G (2020). A WEE1 family business: regulation of mitosis, cancer progression, and therapeutic target. J Hematol Oncol.

[22] Liu Y, Chen X, Hu Q (2018). Resistance to radiotherapy in lung cancer. Int J Clin Exp Med.

[23] Klein C, Dokic I, Mairani A (2017). Overcoming hypoxia-induced tumor radioresistance in non-small cell lung cancer by targeting DNA-dependent protein kinase in combination with carbon ion irradiation. Radiat Oncol.

[24] Carlson DJ, Stewart RD, Semenenko VA (2006). Effects of oxygen on intrinsic radiation sensitivity: a test of the relationship between aerobic and hypoxic linear-quadratic (LQ) model parameters. Med Phys.

[25] Kaplan AR, Glazer PM (2020). Impact of hypoxia on DNA repair and genome integrity. Mutagenesis.

[26] Bindra RS, Schaffer PJ, Meng A (2004). Down-regulation of Rad51 and decreased homologous recombination in hypoxic cancer cells. Mol Cell Biol.

[27] Chan N, Koritzinsky M, Zhao H (2008). Chronic hypoxia decreases synthesis of homologous recombination proteins to offset chemoresistance and radioresistance. Cancer Res.

[28] Meng AX, Jalali F, Cuddihy A (2005). Hypoxia down-regulates DNA double strand break repair gene expression in prostate cancer cells. Radiother Oncol.

[29] Wozny AS, Alphonse G, Cassard A (2020). Impact of hypoxia on the double-strand break repair after photon and carbon ion irradiation of radioresistant HNSCC cells. Sci Rep.

[30] Oliveira PH, Boura JS, Abecasis MM (2012). Impact of hypoxia and long-term cultivation on the genomic stability and mitochondrial performance of ex vivo expanded human stem/stromal cells. Stem Cell Res.

[31] Hauth F, Toulany M, Zips D, Menegakis A (2017). Cell-line dependent effects of hypoxia prior to irradiation in squamous cell carcinoma lines. Clin Transl Radiat Oncol.

[32] Jansen J, Vieten P, Pagliari F (2021). A novel analysis method for evaluating the interplay of oxygen and ionizing radiation at the gene level. Front Genet.

[33] Bee L, Fabris S, Cherubini R, Mognato M, Celotti L (2013). The efficiency of homologous recombination and non-homologous end joining systems in repairing double-strand breaks during cell cycle progression. PLoS ONE.

[34] Chao HX, Poovey CE, Privette AA (2017). Orchestration of DNA damage checkpoint dynamics across the human cell cycle. Cell Syst.

[35] Rothkamm K, Krüger I, Thompson LH, Löbrich M (2003). Pathways of DNA double-strand break repair during the mammalian cell cycle. Mol Cell Biol.

[36] Toulany M (2022). Targeting K-Ras-mediated DNA damage response in radiation oncology: current status, challenges and future perspectives. Clin Transl Radiat Oncol.

[37] Wang M, Kern AM, Hülskötter M (2014). EGFR-mediated chromatin condensation protects KRAS-mutant cancer cells against ionizing radiation. Cancer Res.

[38] Gurtner K, Kryzmien Z, Koi L (2020). Radioresistance of KRAS/TP53-mutated lung cancer can be overcome by radiation dose escalation or EGFR tyrosine kinase inhibition in vivo. Int J Cancer.

[39] Zhu DQ, Liu Y, Yu ZJ (2022). The diverse analysis identifies mutated kras associated with radioresistance in non-small cell lung cancer. World J Oncol.

[40] Blanco R, Iwakawa R, Tang M (2009). A gene-alteration profile of human lung cancer cell lines. Hum Mutat.

[41] Korrodi-Gregório L, Soto-Cerrato V, Vitorino R (2016). From proteomic analysis to potential therapeutic targets: functional profile of two lung cancer cell lines, A549 and SW900, widely studied in pre-clinical research. PLoS ONE.

[42] Markovic J, García-Gimenez JL, Gimeno A (2010). Role of glutathione in cell nucleus. Free Radic Res.

[43] Franco R, Cidlowski JA (2009). Apoptosis and glutathione: beyond an antioxidant. Cell Death Differ.

[44] Dalton TP, Chen Y, Schneider SN (2004). Genetically altered mice to evaluate glutathione homeostasis in health and disease. Free Radic Biol Med.

[45] Solis LM, Behrens C, Dong W (2010). Nrf2 and Keap1 abnormalities in non-small cell lung carcinoma and association with clinicopathologic features. Clin Cancer Res.

[46] Singh A, Bodas M, Wakabayashi N (2010). Gain of Nrf2 function in non-small-cell lung cancer cells confers radioresistance. Antioxid Redox Signal.

[47] Jeong Y, Hoang NT, Lovejoy A (2017). Role of KEAP1/NRF2 and TP53 mutations in lung squamous cell carcinoma development and radiation resistance. Cancer Discov.

[48] Guzy RD, Schumacker PT (2006). Oxygen sensing by mitochondria at complex III: the paradox of increased reactive oxygen species during hypoxia. Exp Physiol.

[49] Hernansanz-Agustín P, Izquierdo-Álvarez A, Sánchez-Gómez FJ (2014). Acute hypoxia produces a superoxide burst in cells. Free Radic Biol Med.

[50] Yang F, Yi M, Liu Y (2018). Glutaredoxin-1 silencing induces cell senescence via p53/p21/p16 signaling axis. J Proteome Res.

[51] Bourgeais J, Ishac N, Medrzycki M (2017). Oncogenic STAT5 signaling promotes oxidative stress in chronic myeloid leukemia cells by repressing antioxidant defenses. Oncotarget.

[52] Chen X, Lv Q, Hong Y (2017). IL-1β maintains the redox balance by regulating glutaredoxin 1 expression during oral carcinogenesis. J Oral Pathol Med.

