# Supplementary material for: Radio-resistance of hypoxic tumors: exploring the effects of oxygen and X-ray radiation on non-small lung cancer cell lines
Source: Radiat Oncol. 2023 May 12;18:81. doi: 10.1186/s13014-023-02275-8 (PMC10182694; doi:10.1186/s13014-023-02275-8)
Supplement: Supplementary file 1 — Additional file 1. Supplementary Materials and Methods. [file 13014_2023_2275_MOESM1_ESM.docx]

Additional File 1

Supplementary Materials and Methods

**Clonogenic assay**

In normoxia, after irradiation, cells were seeded, including the control flask (0 Gy), at increasing densities proportionally to the increasing X-ray dose (0.2x10^3^, 0.222x10^3^, 0.8x10^3^, 3x10^3^, 8x10^3^, for 0, 2, 4, 6 and 8 Gy, respectively). In hypoxic conditions, cells were placed in the hypoxic chamber at 1% overnight, and seeded at densities of 3x10^5^ in T75 flasks. Cells were then maintained at 0.1% O_2_ overnight. Afterwards, the flasks were tightly sealed and put inside hermetically sealed Ziploc bags to ensure the hypoxic environment was maintained during IR (4, 8, 12 and 16 Gy). Subsequently, cells were seeded in T25 cm^2^ flasks at different concentrations, including the control flask (0 Gy) (0.2 x10^3^, 0.222x10^3^, 0.5x10^3^, 0.8x10^3^, 3x10^3^, for 0, 4, 8, 12 and 16 Gy, respectively). After a variable time ranging from 10 to 14 days depending on the cell line and the O_2_ conditions, colonies were fixed and stained with Crystal violet (a threshold of 50 cells per colony was used). The survival fraction for each cell line was calculated using the following formula S(D)=n(D)/N(D) x 1/PE, where N is the initial number of cells seeded, n is the number of colonies counted post-treatment and PE is the plating efficiency.

**Nuclear and cytosolic H_2_O_2_ detection**

For measuring nuH_2_O_2_, cells were seeded in 100 mm petri dishes (Greiner Bio-One) at a density of 1.0x10^6^ the day before IR both in normoxia and in hypoxia. To detect cyH_2_O_2_, 8x10^5^ cells were seeded in 60 mm petri dishes (Greiner Bio-One) the day before IR. After the two time points (30 min or 24 hrs post-IR) cells were stained with 10 μM of NucPE1 or 40 μM of PY1-ME, in HBSS solution and incubated for 20 min under a low oxygenated environment (1% O_2_) for normoxia and 0.1% O_2_ in hypoxia. Of note, cells in normoxia were stained in mild hypoxic conditions to prevent the excessive oxidation of the dyes due to the O_2_, so to allow a better comparison between the normoxic and hypoxic samples. Subsequently, cells were washed with PBS and detached using TrypLE Express (Thermo Fisher Scientific). For the nuH_2_O_2_, cells were resuspended in cold 0.1% NP-40 in PBS and dissociated by pipetting up and down 5 times. Then, the solutions were centrifuged at 10000 rpm at 4°C for 10 seconds. In the subsequent steps, the pellets were again resuspended in 0.1% NP-40, centrifuged and resuspended in ice-cold PBS for the last centrifugation. Finally, the pellets (nuclei) were resuspended in ice-cold PBS and transferred in pre-chilled FACS tubes and immediately acquired at a FACS Canto ™ II flow cytometer (Becton Dickinson).

For the cyH_2_O_2_, after detachment, cells were washed with cold PBS, centrifuged 1200 rpm for 5 min and transferred to cold FACS tubes.

NucPE1 and PY1-ME signals were both excited with a 488 nm laser and the fluorescence emissions collected at 530/30 nm.

**RNA isolation and Quantitative real time-polymerase chain reaction (RT-qPCR)**

RNA was isolated from non-irradiated and irradiated normoxic and hypoxic cell lines 24 hrs after IR using High Pure Isolation Kit (Roche, Germany) as per instruction from the manufacturer. Following extraction, RNA amount and purity were evaluated using a Nanodrop ND-1000 (Thermo Fisher Scientific) and RNA integrity by running a 1% agarose gel. cDNA was produced by retrotranscribing 1 µg of RNA with RT2 First Strand Kit (Qiagen, Germany). Gene expression was analysed by RT-qPCR. Briefly, a reaction mix containing 20 pmol of the forward and reverse primer pairs, Power SYBR Green PCR Master mix (Thermo Fischer Scientific) and nuclease free water (Thermo Fischer Scientific) was used to amplify 25 ng of cDNA. The RT-qPCR protocol consisted of the following cycles: 1 cycle at 95°C for 10 min, 40 cycles at 95°C for 15 seconds (s) and 60°C for 60 s. The relative gene expression was then normalized to the housekeeping gene HPRT1. The ΔΔCt method was used to perform all data analysis. The results are presented as ΔΔCt values normalized to 0 Gy normoxic samples and HPRT1 gene was used as internal reference gene.

The sequences of the primers used were designed on the CDS of the target genes and are listed below.

| **Name** | **Primer Forward** *5’*🡪*3’* | **Primer Reverse** *5’*🡪*3’* | **NCBI Reference Sequence** |
| --- | --- | --- | --- |
| HPRT1 | TTGACACTGGCAAAACAATG | GTCCTTTTCACCAGCAAGC | NM_000194.3 |
| RAD50 | GGGAAAGACGACCATCATTG | TCTTGAGCAACCTTGGGATC | NM_005732.3 |
| XRCC5 | GTGGGGATCAGTATCAGAAC | GAAGTCAGCCTGTTGAGAAC | NM_021141.4 |
| XRCC6 | GCTTCTGCCTAGCGATACC | TACCAACGGCTTGAAACCC | NM_001469.4 |
| DNA-PKcs | CTCTGTGTGAACTGGTTGCG | GAGGAGGGAAGCTCTTGGTC | NM_006904.7 |
| DCLRE1C | CATGTTGTGTGGCTGAACTG | GATCTGAGTGTTGCGGTCTG | NM_001033855.3 |
| LIG4 | CTGCCCCAAAGATGAAGAAAG | CAATTCTGTTCTCCAGGTCAG | NM_002312.3 |
| RAD51 | CACCGCCCTTTACAGAACAG | TCCACTTGAGCTACCACCTG | NM_002875.5 |
| RAD52 | GTGCTACATTGAGGGTCATCG | AATCCACATTCTGCTGCGTG | NM_134424.3 |
| BRCA1 | TTCACCAACATGCCCACAG | ATCTGGCTGCACAACCACA | NM_007294.3 |
| BRCA2 | CACCTCTTGAAGCCCCAGAA | AGCGATGATAAGGGCAGAGG | NM_000059.4 |
| WEE1 | TGTGCGACAGACTCCTCAAG | GGCTTCCATGTCTTCACCAC | NM_003390.4 |
| NFE2L2 | CTCCATATCCCATTCCCTGTAG | GAGCAGCCACTTTATTCTTACC | NM_006164.5 |
| CAT | CGTGCTGAATGAGGAACAG | GACCGCTTTCTTCTGGATG | NM_001752.4 |
| GPX1 | CCCAAGCTCATCACCTGGTC | TGTCAATGGTCTGGAAGCGG | NM_000581.4 |
| GPX4 | GAA ATGCCATCAAGTGGAAC | CAGGTCCTTCTCTATCACCA | NM_002085.5 |
| GSR | GAAGAAAAGGTGGTTGGGATC | GAATGGCGACTGTGTTGTC | NM_000637.5 |
| GLRX | ACAGCCACCAACCACACT | GCAAAGAGACTAGATCACTGC | NM_002064.3 |
