# Supplementary material for: Radio-resistance of hypoxic tumors: exploring the effects of oxygen and X-ray radiation on non-small lung cancer cell lines
Source: Radiat Oncol. 2023 May 12;18:81. doi: 10.1186/s13014-023-02275-8 (PMC10182694; doi:10.1186/s13014-023-02275-8)
Supplement: Supplementary file 2 — Additional file 2. Additional Tables 1-6 reporting values of cell survival fractions, alpha and beta, doses at 10% of cell survival, Cell cycle data, NucPE1 and PY1-ME fluorescence signals. [file 13014_2023_2275_MOESM2_ESM.docx]

Additional file 2

**Additional Table 1. Cell survival fraction (SF±SD) following photon irradiation with varying doses (Gy).**

| SF ± SD (%) after IR | Cell line | 2 Gy | 4 Gy | 6 Gy | 8 Gy |
| --- | --- | --- | --- | --- | --- |
| Normoxia | H460 | 66.5 ± 0.4 | 15.4 ± 3.2 | 3.3 ± 1.1 | 0.4 ± 0.2 |
|  | A549 | 53.3 ± 8.7 | 21.8 ± 10.0 | 6.8 ± 2.7 | 1.5 ± 0.6 |
|  | Calu-1 | 53.0 ± 19.0 | 22.1 ± 10.7 | 5.6 ± 1.1 | 1.0 ± 0.2 |
|  |  | **4 Gy** | **8 Gy** | **12 Gy** | **16 Gy** |
| Hypoxia | H460 | 43.9 ± 21.2 | 17.9 ± 8.2 | 9.5 ± 2.6 | 1.8 ± 0.9 |
|  | A549 | 43.3 ± 6.8 | 8.5 ± 4.8 | 14.7 ± 8.7 | 0.5 ± 0.4 |
|  | Calu-1 | 49.9 ± 21.2 | 17.4 ± 11.0 | 5.7 ± 2.1 | 2.1 ± 2.3 |

**Additional Table 2. Alpha (α) and Beta (β) values determined by fitting of the linear quadratic model to the clonogenic data and using the supplementary PE-based MS excel tool according to Nature Protocols [**1**].**

|  | Cell line | Alpha (α)±SD | Beta (β)±SD | | Ratio (α/β) ±SD |
| --- | --- | --- | --- | --- | --- |
| Normoxia | H460 | 0.156 ± 0.047 | | 0.0606 ± 0.0069 | 2.26 ± 0.722 |
|  | A549 | 0.234 ± 0.006 | | 0.0364 ± 0.0009 | 6.43 ± 0.231 |
|  | Calu-1 | 0.202 ± 0.013 | | 0.0463 ± 0.0019 | 4,36 ± 0.336 |
| Hypoxia | H460 | 0.177 ± 0.009 | | 0.0043 ± 0.00064 | 41.01 ± 6.392 |
|  | A549 | 0.139 ± 0.111 | | 0.0107 ± 0.00807 | 12.99 ± 14.25 |
|  | Calu-1 | 0.221 ± 0.042 | | 0.0015 ± 0.0003 | 145.97 ± 293.11 |

**Additional Table 3. Dose at 10% of cell survival based on the average SF data obtained from the clonogenic assays.**

| Dose at 10% SF (D10) ±SD | Normoxia | Hypoxia | OER |
| --- | --- | --- | --- |
| H460 | 4.754 ± 0.337 | 10.368 ± 0.427 | 2.18 ± 0.179 |
| A549 | 5.363 ± 0.067 | 9.536 ± 3.749 | 1.78 ± 0.699 |
| Calu-1 | 5.204 ± 0.125 | 9.780 ± 1.993 | 1.88 ± 0.386 |

**Additional Table 4. Cell cycle data obtained 24 hrs after photon irradiation. Data are presented as mean±SD.**

| **H460: 24 hrs post-IR** | | | | | |
| --- | --- | --- | --- | --- | --- |
| **% of cells in cell phase** | **Phase** | **0 Gy** | **2Gy** | **4Gy** | **8Gy** |
| **Normoxia** | G0-G1 | 66.7 ± 0.81 | 74.3 ± 2.04 | 81 ± 0.33 | 55.2 ± 4.01 |
|  | S | 13.5 ± 0.54 | 8.9 ± 1.65 | 6 ± 0.16 | 3 ± 0.19 |
|  | G2 | 19.8 ± 0.37 | 16.8 ± 0.52 | 13 ± 0.31 | 41.8 ± 4.22 |
|  |  |  |  |  |  |
|  | **Phase** | **0 Gy** | **4 Gy** | **8 Gy** | **16 Gy** |
| **Hypoxia** | G0-G1 | 77.2 ± 0.29 | 75.5± 1.43 | 64.6 ± 1.24 | 57.3± 0.62 |
|  | S | 8.3 ± 0.39 | 11.5 ± 1.53 | 7.7 ± 1.11 | 9.7 ± 0.43 |
|  | G2 | 14.5 ± 0.66 | 13 ± 0.41 | 27.7 ± 1.51 | 33± 1.34 |
| **A549: 24 hrs post-IR** | | | | | |
| **% of cells in cell phase** | **Phase** | **0 Gy** | **2Gy** | **4Gy** | **8Gy** |
| **Normoxia** | G0-G1 | 50.6 ± 0.66 | 53.1 ± 0.78 | 57.7 ± 2.99 | 56.2 ± 1.55 |
|  | S | 12.1 ± 0.37 | 12.3 ± 0.34 | 10.0 ± 1.07 | 8.1 ± 1.21 |
|  | G2 | 37.3 ± 0.41 | 34.6 ± 0.43 | 32.3 ± 3.96 | 35.6 ± 2.67 |
|  |  |  |  |  |  |
|  | **Phase** | **0 Gy** | **4 Gy** | **8 Gy** | **16 Gy** |
| **Hypoxia** | G0-G1 | 69.2 ± 0.48 | 56.2 ± 0.69 | 52.0 ± 0.83 | 41.5 ± 0.73 |
|  | S | 5.9 ± 0.19 | 5.7 ± 0.17 | 6 ± 0.87 | 11.5 ± 1.01 |
|  | G2 | 24.9 ± 0.39 | 38.1 ± 0.71 | 42.0 ± 1.61 | 47 ± 1.69 |
| **Calu-1: 24 hrs post-IR** | | | | | |
| **% of cells in cell phase** | **Phase** | **0 Gy** | **2Gy** | **4Gy** | **8Gy** |
| **Normoxia** | G0-G1 | 64.5 ± 0.45 | 69.3 ± 0.05 | 68± 1.01 | 49.4 ± 3.09 |
|  | S | 10.0 ± 0.75 | 9.4 ± 0.42 | 7 ± 0.41 | 5.5 ± 0.16 |
|  | G2 | 25.3 ± 0.75 | 21.5 ± 0.47 | 25 ± 0.99 | 45.1 ± 3.11 |
|  |  |  |  |  |  |
|  | **Phase** | **0 Gy** | **4 Gy** | **8 Gy** | **16 Gy** |
| **Hypoxia** | G0-G1 | 82 ± 2.03 | 76 ± 4.57 | 75.5 ± 1.59 | 71.6 ± 1.63 |
|  | S | 8.3 ± 1.36 | 8.4 ± 2.73 | 7 ± 1.69 | 7.8 ± 1.10 |
|  | G2 | 9.7 ± 0.65 | 15.6 ± 2.00 | 17.5 ± 2.66 | 20.6 ± 0.70 |

**Additional Table 5. NucPE1 fluorescence signal data normalized to 0 Gy control sample.**

| **NucPE1** | **NORMOXIA** | | | | | | |
| --- | --- | --- | --- | --- | --- | --- | --- |
| **30 min** | **Dose (Gy)** | **H460 ± SEM** | | **A549 ± SEM** | | **Calu-1 ± SEM** | |
|  | 0 | 1.000 | 0.102 | 1.000 | 0.253 | 1.000 | 0.053 |
|  | 2 | 2.797 | 0.465 | 1.091 | 0.212 | 1.320 | 0.064 |
|  | 4 | 3.049 | 0.459 | 2.400 | 0.456 | 1.720 | 0.106 |
|  | 8 | 2.812 | 0.698 | 3.522 | 0.631 | 2.305 | 0.128 |
|  |  |  | . |  |  |  |  |
| **24 hrs** | **Dose (Gy)** | **H460 ± SEM** | | **A549 ± SEM** | | **Calu-1 ± SEM** | |
|  | 0 | 1.000 | 0.113 | 1.000 | 0.058 | 1.000 | 0.188 |
|  | 2 | 0.706 | 0.228 | 0.901 | 0.090 | 1.573 | 0.224 |
|  | 4 | 0.658 | 0.133 | 1.503 | 0.103 | 1.862 | 0.276 |
|  | 8 | 1.340 | 0.449 | 2.326 | 0.136 | 2.819 | 0.415 |
|  |  |  |  |  |  |  |  |
|  | **HYPOXIA** | | | | | | |
| **30 min** | **Dose (Gy)** | **H460 ± SEM** | | **A549 ± SEM** | | **Calu-1 ± SEM** | |
|  | 0 | 1.000 | 0.059 | 1.000 | 0.123 | 1.000 | 0.099 |
|  | 4 | 1.227 | 0.071 | 0.798 | 0.072 | 0.919 | 0.078 |
|  | 8 | 0.847 | 0.135 | 0.901 | 0.091 | 1.201 | 0.088 |
|  | 16 | 0.746 | 0.031 | 0.491 | 0.060 | 0.706 | 0.075 |
|  |  |  |  |  |  |  |  |
| **24 hrs** | **Dose (Gy)** | **H460 ± SEM** | | **A549 ± SEM** | | **Calu-1 ± SEM** | |
|  | 0 | 1.000 | 0.174 | 1.000 | 0.140 | 1.000 | 0.171 |
|  | 4 | 0.788 | 0.161 | 1.127 | 0.169 | 1.568 | 0.210 |
|  | 8 | 0.930 | 0.134 | 1.150 | 0.115 | 2.684 | 0.326 |
|  | 16 | 0.825 | 0.103 | 1.009 | 0.116 | 2.460 | 0.551 |

**Additional Table 6.** **PY1-ME fluorescence signal data normalized to 0 Gy control sample.**

| **PY1-ME** | **NORMOXIA** | | | | | | |
| --- | --- | --- | --- | --- | --- | --- | --- |
| **30 min** | **Dose (Gy)** | **H460 ± SEM** | | **A549 ± SEM** | | **Calu-1 ± SEM** | |
|  | 0 | 1.000 | 0.103 | 1.000 | 0.474 | 1.000 | 0.105 |
|  | 2 | 1.587 | 0.154 | 1.287 | 0.640 | 0.849 | 0.072 |
|  | 4 | 1.614 | 0.185 | 0.991 | 0.419 | 0.799 | 0.165 |
|  | 8 | 1.871 | 0.320 | 1.233 | 0.571 | 0.983 | 0.091 |
|  |  |  |  |  |  |  |  |
| **24 hrs** | **Dose (Gy)** | **H460 ± SEM** | | **A549 ± SEM** | | **Calu-1 ± SEM** | |
|  | 0 | 1.000 | 0.022 | 1.000 | 0.126 | 1.000 | 0.198 |
|  | 2 | 1.285 | 0.133 | 0.899 | 0.150 | 1.029 | 0.233 |
|  | 4 | 1.514 | 0.053 | 1.023 | 0.151 | 0.947 | 0.183 |
|  | 8 | 1.909 | 0.113 | 1.378 | 0.446 | 1.646 | 0.254 |
|  |  |  |  |  |  |  |  |
|  | **HYPOXIA** | | | | | | |
| **30 min** | **Dose (Gy)** | **H460 ± SEM** | | **A549 ± SEM** | | **Calu-1 ± SEM** | |
|  | 0 | 1.000 | 0.037 | 1.000 | 0.241 | 1.000 | 0.421 |
|  | 4 | 0.243 | 0.032 | 1.430 | 0.711 | 0.463 | 0.153 |
|  | 8 | 0.273 | 0.069 | 1.201 | 0.280 | 0.615 | 0.198 |
|  | 16 | 0.193 | 0.089 | 1.132 | 0.493 | 0.322 | 0.114 |
|  |  |  |  |  |  |  |  |
| **24 hrs** | **Dose (Gy)** | **H460 ± SEM** | | **A549 ± SEM** | | **Calu-1 ± SEM** | |
|  | 0 | 1.000 | 0.195 | 1.000 | 0.258 | 1.000 | 0.054 |
|  | 4 | 0.466 | 0.110 | 0.336 | 0.078 | 0.486 | 0.090 |
|  | 8 | 0.203 | 0.029 | 0.262 | 0.087 | 0.327 | 0.038 |
|  | 16 | 0.102 | 0.031 | 0.126 | 0.023 | 0.195 | 0.028 |

1. Brix N, Samaga D, Belka C. et al. Analysis of clonogenic growth in vitro. Nat Protoc 16, 4963–4991 (2021).
