# Supplementary figures and images for: Radio-resistance of hypoxic tumors: exploring the effects of oxygen and X-ray radiation on non-small lung cancer cell lines
Source: Radiat Oncol. 2023 May 12;18:81. doi: 10.1186/s13014-023-02275-8 (PMC10182694; doi:10.1186/s13014-023-02275-8)

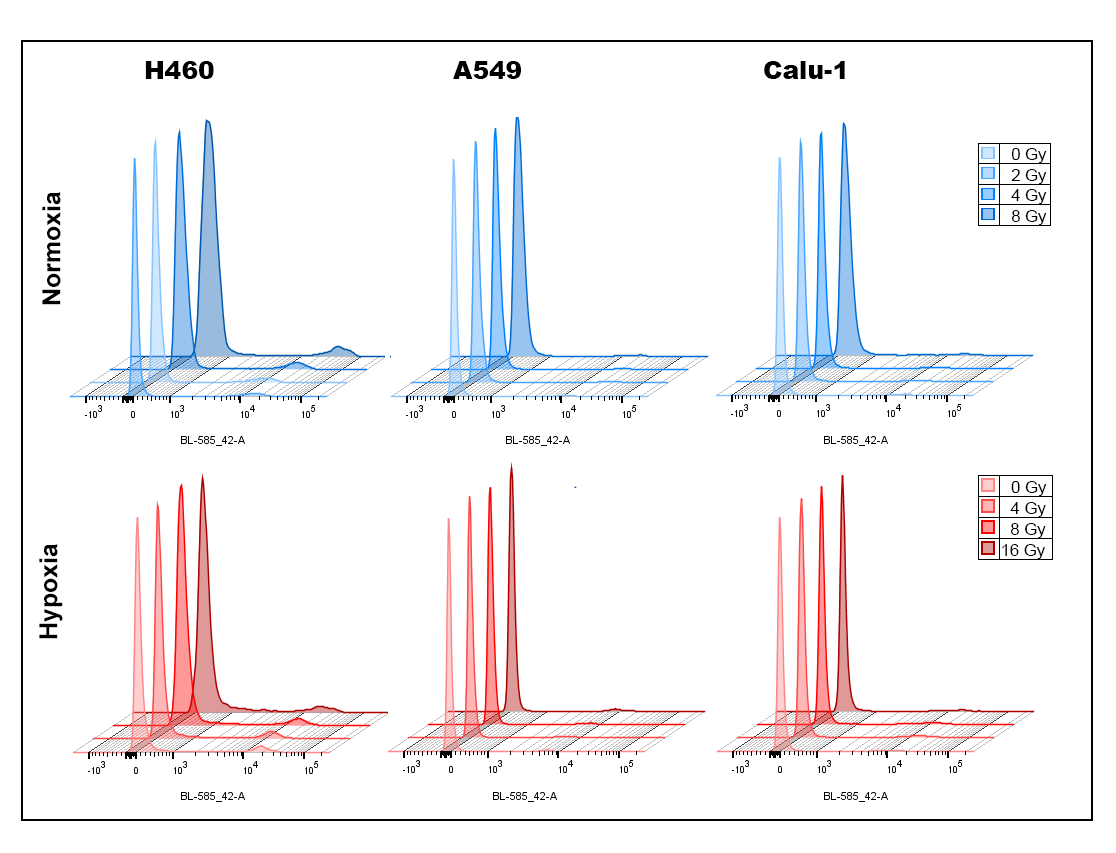

Supplement: Supplementary file 3 — Additional file 3. Fig. 1: Representative histograms of cell death after IR in normoxia and hypoxia as assessed by Propidium Iodide staining and subsequent FACS analysis [file 13014_2023_2275_MOESM3_ESM.tif]

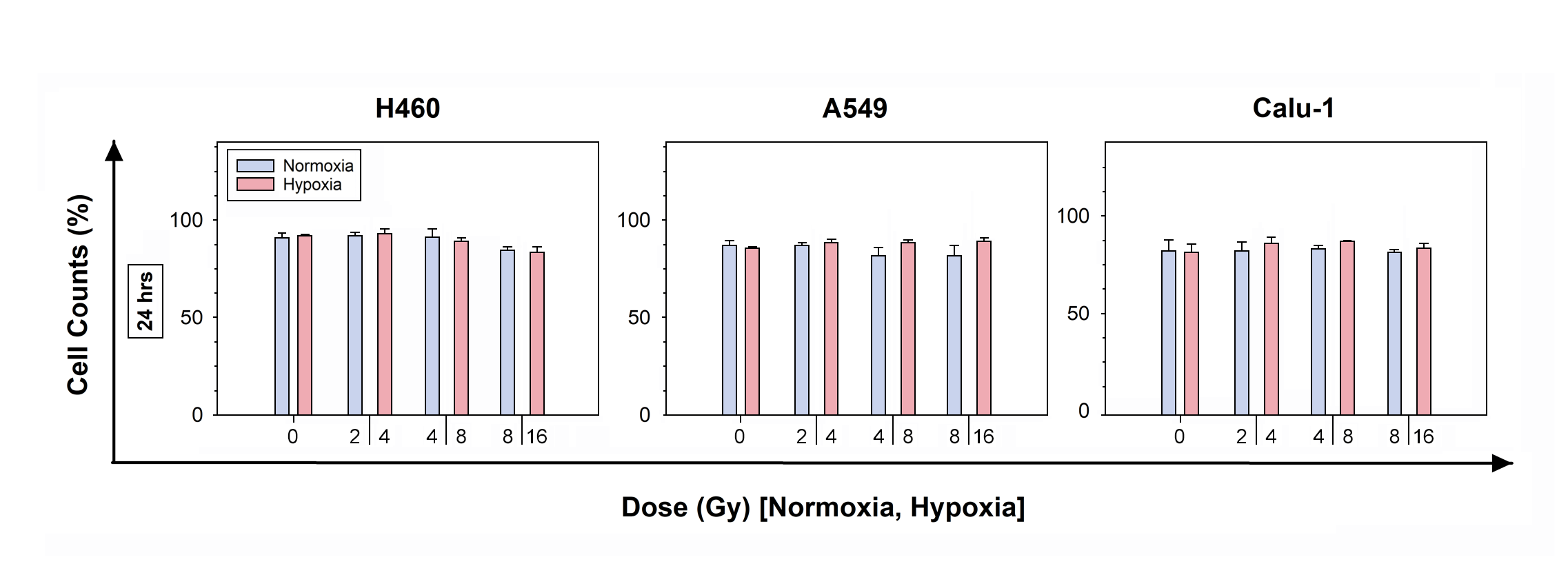

Supplement: Supplementary file 4 — Additional file 4. Fig. 2: Cell growth at 24 hrs after IR in normoxia and hypoxia as assessed by Trypan blue assay. Data are shown as mean±SD. [file 13014_2023_2275_MOESM4_ESM.tif]
